# Supplementary material for: Decoupled choice-driven and stimulus-related activity in parietal neurons may be misrepresented by choice probabilities
Source: Nat Commun. 2017 Sep 28;8:715. doi: 10.1038/s41467-017-00766-3 (PMC5620044; doi:10.1038/s41467-017-00766-3)
Supplement: Supplementary file 1 — Supplementary Information [file 41467_2017_766_MOESM1_ESM.pdf]

File Name: Supplementary Information
Description: Supplementary Figures and Supplementary Notes

File Name: Peer Review File

SUPPLEMENTARY NOTES

Supplementary Note 1: Partial correlation simulation

To provide insight and intuition regarding the partial correlation analysis, we simulated neuronal data (300 cells) such that each cell's FR responses comprised independent contributions of heading and choice (Supplementary Figure 1). First, a heading vector was created with the actual headings used in the experiment: $\boldsymbol{h} = [-12°;\ -6°;\ -1.5°;\ 0°;\ 1.5°;\ 6°; 12°]$. Then, choices (coded as +1 for rightward and -1 for leftward choices) were simulated using binomial random variates, drawn with heading-specific probabilities from a cumulative Gaussian psychometric function ($\mu = 0°$ and $\sigma = 3°$). This resulted in a choice matrix $\boldsymbol{C_i}$ with elements $C_i(j,l)$, where i is the cell number (i = 1 to 300), j is the heading index (j = 1 to 7, representing the headings from left to right, respectively), and l is the repetition index (ten stimulus repetitions were simulated per heading, l = 1 to 10). The heading vector $\boldsymbol{h}$ was then normalized by its root mean square value (RMS; comparable to the choice parameter which already had RMS = 1) and concatenated ten times (for the 10 repeats), to create a normalized heading matrix $\boldsymbol{H}$ (with elements $H(j,l)$). While the choice matrix was simulated for each cell (representing different experimental sessions), the heading matrix (which represents the stimulus) was constant.

Next, heading and choice beta coefficients were generated, for each model neuron, using two uncorrelated Gaussians, βh_i and βc_i, respectively (each $\sim N(0,1)$), and trial-by-trial heading and choice contributions were calculated by multiplying the normalized heading and choice parameters ($H(j,l)$ and $C_i(j,l)$) by their respective beta coefficients. Simulated FR responses were then calculated by a linear combination of the heading and choice contributions (together, the 'signal', with combined $\sigma_S = \sqrt{2}$) with the addition of random Gaussian noise $N_i(j,l) \sim N(0,\sigma_N)$, as follows:

$$FR_i(j,l) = \beta h_i \cdot H(j,l) + \beta c_i \cdot C_i(j,l) + N_i(j,l) \qquad \text{(Suppl. Eq. 1)}$$

The level of added noise was adjusted to attain three levels of signal to noise ratio (SNR): ~20dB (high SNR; $\sigma_N = 0.1\sqrt{2}$), ~0dB (medium SNR; $\sigma_N = \sqrt{2}$) and ~ -20dB (low SNR; $\sigma_N = 10\sqrt{2}$). These three SNR levels were simulated to explore the joint distribution of heading and choice partial correlations for different levels of noise (Supplementary Figure 1, left column), and to show that when noise is dominant (low SNR) a spurious relationship between the partial correlation parameters can arise (described further below).

For high SNR (Supplementary Figure 1A) strong heading and choice components are observed (clusters of data around the corners). For medium SNR (Supplementary Figure 1B), a balanced spread of the data in the partial correlation plane is seen, without any specific trend (as expected, given that βh_i and βc_i were generated from two uncorrelated Gaussian distributions). We further used the medium SNR data (because of their even spread across the plane) to demonstrate that CPs can be biased even when heading and choice components are completely uncorrelated. For this analysis, we calculated CPs in the same manner as shown in Figure 3 of the main manuscript (Supplementary Figure 1C).

Here we see that CPs are predominantly high (CP > 0.5; large green areas marked by white dashed lines), with the mean CP across the plane (0.63) significantly greater than 0.5 (two-tailed t-test; $p < 10^{-12}$). While one may have expected a balanced distribution of CPs – high (CP > 0.5) in the top-right and bottom-left quadrants, and low (CP < 0.5) in the top-left and bottom-right quadrants – all data in the vicinity of the vertical axis (as well as some data in the top-left and bottom-right quadrants) have high CPs (green). This is because the choice component of response dominates the tuning curve that is used to define the "preferred heading" when CPs are calculated. In such cases, CPs are guaranteed to be >0.5 even when response components related to heading and choice are opposite in sign. This result is important since it shows that uncorrelated

heading and choice signals can yield CPs (as traditionally calculated) that are biased toward large values (> 0.5).

When heading and choice contributions are of equal magnitude but opposite in sign (along y = –x), CPs will more likely (correctly) reflect the fact that heading and choice signals have opposite effects on FRs (CP < 0.5). This is because the tuning preference used to calculate a cell's CP was estimated from a regression of FRs against headings, not choice. Take for example a case of two headings (±h). The FRs grouped by heading will (by definition) always comprise responses only to +h or –h, respectively. However, in each of these groups, the choices will be mixed (albeit more rightward choices for +h and more leftward choices for –h). Thus, the regression used to calculate tuning preference for CPs (FRs vs. heading) has an implicit bias to expose stimulus tuning and average out choice tuning. Therefore, when heading and choice contributions are of equal magnitude but opposite sign, the regression for CPs will be characterized primarily by the heading responses and CPs will be <0.5. Hence, both for the simulations in Supplementary Figure 1C, as well as the data in Figure 3, the diagonal dashed line, which marks the border between positive and negative CPs, does not lie perfectly along the diagonal. However, as choice signals become slightly stronger than heading tuning, they dominate the regression of FR vs. heading, and thus dictate the tuning preference used for CP calculation, leading to CPs > 0.5 even for heading and choice contributions of opposite sign.

To see what the scatter of partial correlations would look like if response contributions from heading and choice were correlated, we also simulated (medium SNR) data with correlated heading and choice coefficients (ρ=0.8). Not surprisingly, we see that the heading and choice partial correlations form a positive relationship (Supplementary Figure 1D, left). Namely, if the underlying response components are correlated, then also their unique contributions (the heading and choice partial correlations) will be correlated. This exposes a nuance of the partial correlation

comparison – heading and choice partial correlations reflect the unique contribution of one parameter to the FR by removing the effects of the other. The unique effects can then be studied and compared in order to assess whether they themselves are related.

Supplementary Figure 1E shows that low SNR data can lead to a spurious negative relationship between the partial correlation parameters. This is because a roughly flat FR response (red and purple examples in Supplementary Figure 1F) can be fit by opposite effects of heading and choice, but not by effects of the same sign, which would necessarily sum to a non-flat FR function (blue example cell). Thus, highly noisy data (low SNR) can bias the relationship between heading and choice partial correlations to have a negative correlation. Accordingly, very noisy data should not be included in this analysis. To address this issue, neurons were prescreened using a 2-way ANOVA (heading and choice) and only those which showed a significant main effect of heading or choice ($p<0.05$) were included. ANOVA screening was preferred, rather than linear regression, in order to avoid the assumption of a specific form for the data, and also to avoid circularity (the subsequent analyses involve linear regression).

Note also that the ANOVA screening reduces the effect of random noise by removing a high proportion (~95% for $p<0.05$) of non-responsive cells, relative to tuned cells. However, if the data contain no (or very few) tuned cells, then the problem can persist even after screening. In our data, a large proportion of cells generally passed the ANOVA screening: 46% VIP-visual, 44% MSTd-visual, 31% VIP-vestibular and 14% MSTd-vestibular in the new data set, and 47%, 82%, 55%, 53%, respectively, in the old data set (higher values for the old data are expected due to their pre-recording selection criteria; see Methods). This indicates that the post-ANOVA data comprise mainly genuinely tuned cells. Also, ANOVA screening with $p<0.01$ (passing 32%, 32%, 17%, and 5% of the new data, and 29%, 74%, 41%, 38% of the old data) displayed very similar results to Supplementary Figure 2 (for which $p<0.05$). By contrast, for the simulation in

Supplementary Figure 1E, only two (out of the 300) of the model neurons passed the ANOVA screening with $p<0.01$. Thus, we conclude that the potential problem with noisy data (described above) is not a substantial concern for our neural data after screening by ANOVA.

Supplementary Note 2: Partial correlations, further analysis

An expanded form of the analysis presented in the manuscript (Fig. 2A) is presented in Supplementary Figure 2. Here, type-II regressions are shown separately for each group of data. The results indicate greater dispersion of the data along the choice and heading axes for VIP and MSTd, respectively – corroborating the main findings in the manuscript. However, this partial correlation analysis assumes a linear relationship between FR and heading (within the narrow range of headings tested). Namely, it implicitly assumes a relationship of the form $FR = \beta_{\text{heading}} \cdot heading + \beta_{\text{choice}} \cdot choice + C$, where *heading* and *choice* are the trial-by-trial parameters, β_{heading} and β_{choice} are the cell's respective coefficients, and C is a constant. Since *choice* is a binary parameter of equal magnitude but opposite sign (-1 or 1 for left vs. right choices), non-linear effects need not be considered for the choice component itself. However, including other terms, such as an interaction term between heading and choice ($heading \times choice$) or a nonlinear dependence on heading (such as $heading^2$), may yield a better fit to the firing rate data. If so, this would reduce the amount of variance explained by the linear heading and choice components. Furthermore, unaccounted variance in the heading relationship (if it is nonlinear) could be aliased into a choice effect.

To address this issue, we also fit the data with a more complex model: $FR = \beta_{\text{heading}} \cdot heading + \beta_{\text{choice}} \cdot choice + \beta_{\text{heading\_squared}} \cdot heading^2 + \beta_{\text{interaction}} \cdot heading \times choice + C$, and calculated the partial correlations given all the other terms, namely: $R_{choice} = R(FR, choice \mid heading, heading \times choice, heading^2)$ and $R_{heading} = R(FR, heading \mid$

choice, heading× choice , heading^2*)*. The more complex model must fit the data at least as well as the linear model, since it has more parameters. Although the more complex model improved the fits significantly for a substantial fraction of neurons (Supplementary Figure 3B; model R^2 values should not be confused with the partial correlation R values presented in Supplementary Figure 3A), the variance explained by the linear heading and choice terms was much greater than that explained by the non-linear heading term or the heading-choice interaction term (Supplementary Figure 3C). Importantly, the linear terms (Supplementary Figure 3C) again clearly demonstrate choice-dominance in VIP and heading-dominance in MSTd, even within the more complex model. Finally, calculating partial choice correlations given additional non-linear heading terms, such as *heading*^{3}*or* sigmoid*(heading)* had little effect on the outcome.

Supplementary Note 3: Beta coefficient analysis

For the partial correlation analyses, we used *R* values (correlation coefficients) to summarize the contributions of heading and choice to neural responses. We chose to use those because: i) they carry directional information (unlike R^2) and ii) they are invariant to the magnitude of the stimulus parameters (unlike the beta coefficients of a regression model). While beta coefficients could be used, their magnitudes would depend on how the stimuli are normalized, e.g., using the raw vs. normalized headings would yield different betas (by the factor of normalization). Accordingly, the type of normalization sets the relative importance of heading vs. choice betas – for equal heading and choice betas, the stimulus heading normalized to 1 would contribute the same amount to the FR as a choice (which also has magnitude 1). This is problematic because we do not know how heading and choice variables are actually weighted by individual neurons, hence the normalization of beta coefficients is arbitrary. By contrast, *R* values are resistant to this problem – the same values are obtained irrespective of normalization.

However, *R* values are limited to the range from -1 to 1. Thus, they might have a more ‘compressed’ distribution vs. beta coefficients. To examine this issue, we also performed the same analysis as in Supplementary Figure 2, but using beta coefficients instead of *R* values (Supplementary Figure 4). For this comparison, the headings were normalized by their RMS value (as in the simulations described above) and FRs were normalized by their maximum. Although the heading normalization is arbitrary, the rationale here was to attain the same weight for heading and choice parameters, such that heading and choice betas of equal magnitude would have the same effect on FR. This beta coefficient analysis provided very similar results to the correlation coefficient analysis (compare Supplementary Figures 4 and 2). Additionally, plotting the partial correlations of the simulated data vs. their beta coefficients (calculated in the same manner as described here for the real data) shows that beta coefficients and partial correlations are closely related (Supplementary Figure 1D, right).

SUPPLEMENTARY FIGURES

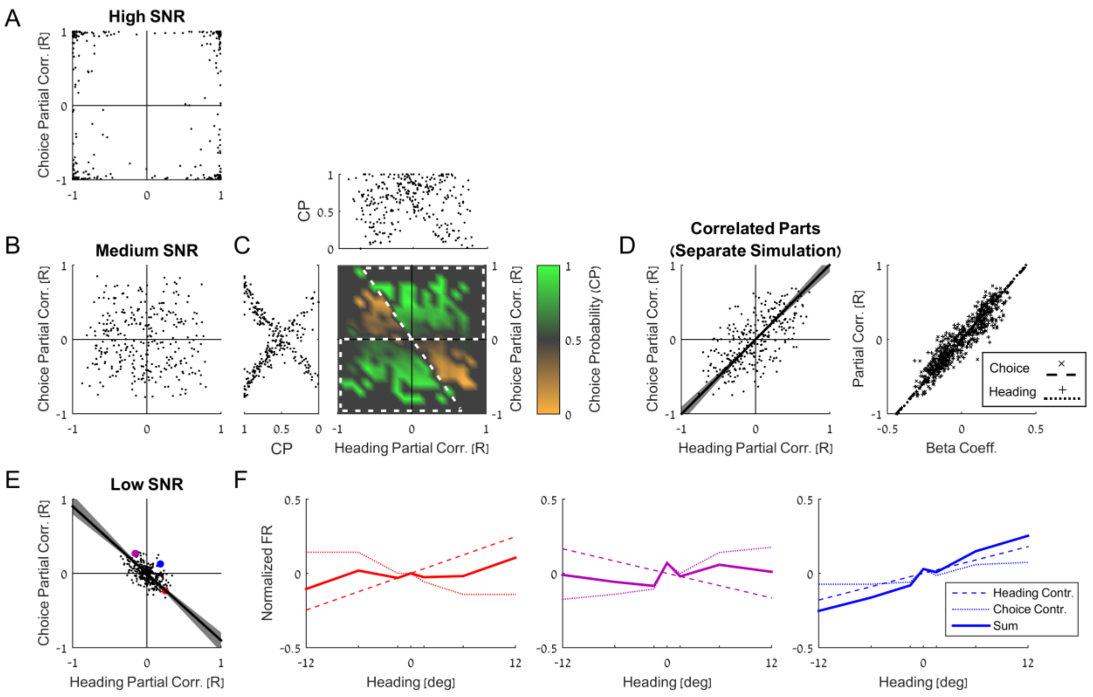

Supplementary Figure 1: Simulation of heading and choice partial correlations. Heading and choice coefficients were generated randomly (and independently, except for D) for N=300 cells. Simulated firing rates (FRs) were then calculated by a linear combination of heading and choice contributions, with added noise. The simulated FRs were then analyzed, using the same partial correlation analysis used in Figure 2A, under conditions of (A) high, (B-D) medium and (E-F) low signal to noise ratios (SNRs). (C) For medium SNR, CPs were calculated and plotted in the same way as in Figure 3. Superimposed white dashed lines emphasize the regions characterized predominantly by high CPs (CP > 0.5; green). Above and to the left of the heat map, marginal plots show the CP scatter vs. heading and choice partial correlations, respectively. For medium SNR the distribution of partial correlations was also plotted for a separate simulation with correlated heading and choice coefficients (ρ=0.8; D, left; we do not show a CP heat map here, since CPs >0.5 are expected due to the correlated coefficients). Here we see a tight relationship between the partial correlation coefficients and the underlying weights (beta coefficients) that were used for the model neurons (D, right). (E) For low SNR, an artificial negative association appears between heading and choice partial correlations. A type-II regression and 95% confidence intervals are marked by the diagonal line and surrounding shaded area in D and E. (F) Heading and choice contributions to FR, and the sum thereof, are presented for three simulated cells (normalized to the maximum absolute simulated FR; there was no constant term, thus this represents the change in FR which can be negative): two with opposing effects of heading and choice (red and purple) and one with positive (same sign) effects (blue). Relatively flat FR functions can result from opposing effects of heading and choice (red or purple), but not positive (same sign) effects, which sum to a steeper slope (blue). The partial correlations of these cells are presented in E, with their respective colors.

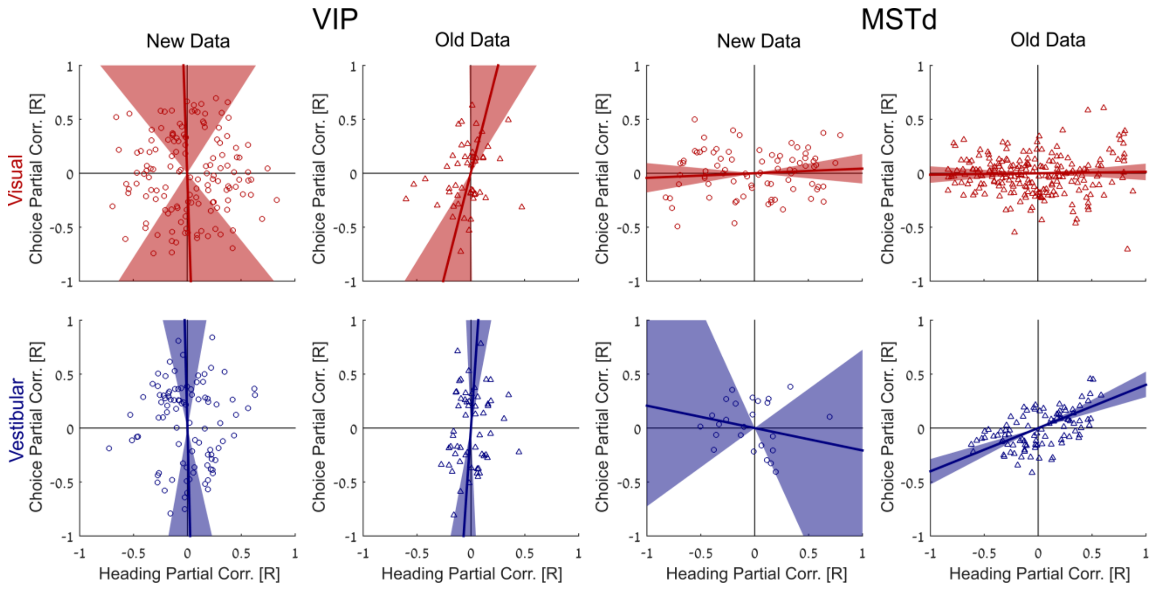

Supplementary Figure 2: Heading and choice partial correlations. The data from Figure 2A are plotted here separately for the visual (red) and vestibular (blue) conditions. Type-II regressions (solid lines) and 95% confidence intervals (shaded regions) are presented for both VIP and MSTd for the new data as well as the old data ('○' and '△' symbols, respectively). In the new MSTd dataset, relatively few cells demonstrated robust vestibular responses (i.e., were multisensory), hence results for the vestibular condition were limited. However, this was supplemented by the additional (old) MSTd data which had more prevalent multisensory responses (see Methods). Cell selection and N values are the same as Figure 2. All p-values for the regressions were > 0.05 except for MSTd vestibular in the old data (bottom right) for which $p = 1.7 \cdot 10^{-8}$.

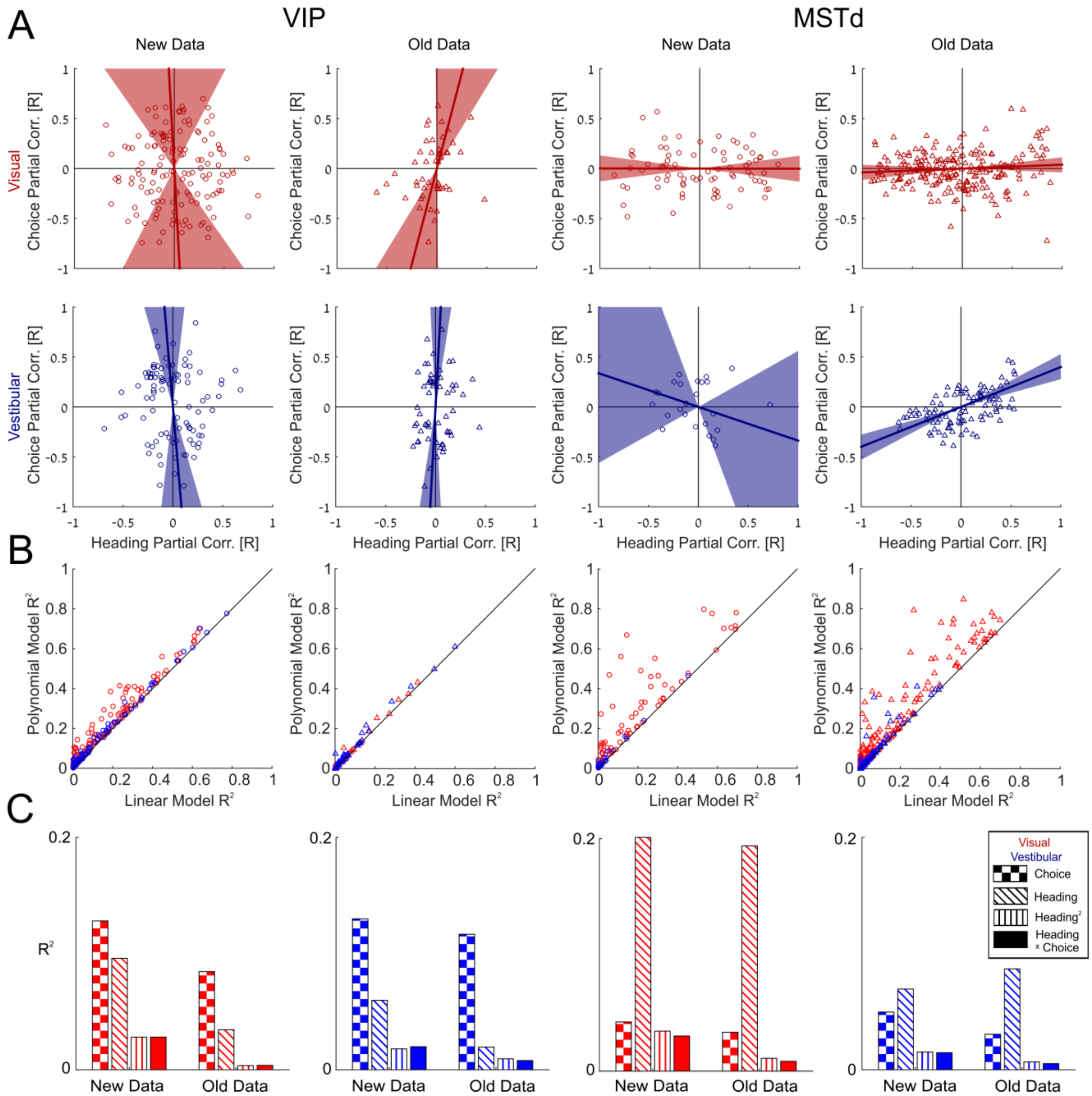

Supplementary Figure 3: Heading and choice partial correlations with a more complex model. (A) Same as Supplementary Figure 2, but calculating the partial regressions given the more complex model. (B) Goodness of fit (R^2) for the more complex vs. linear model fit (not to be confused with the partial correlation R values presented in A). Each point represents the visual (red) and vestibular (blue) fits for a single neuron. (C) The mean R^2 values for each term when fitting the more complex model indicate that the linear heading and choice components account for most of the variance.

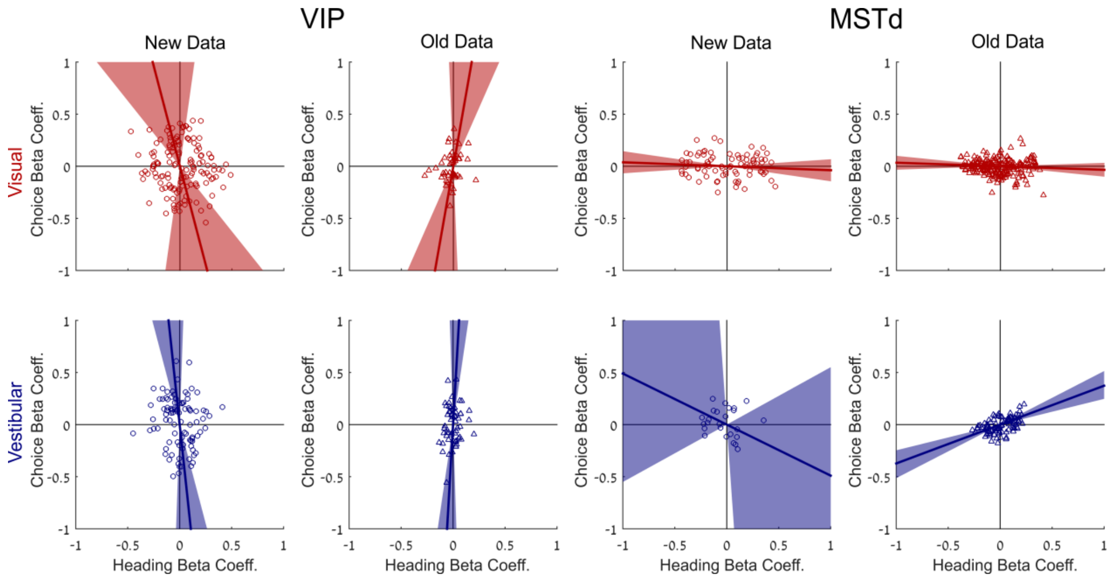

Supplementary Figure 4: Heading and choice beta coefficients. All conventions are the same as Supplementary Figure 2, however, heading and choice beta coefficients are plotted, instead of partial correlation coefficients.

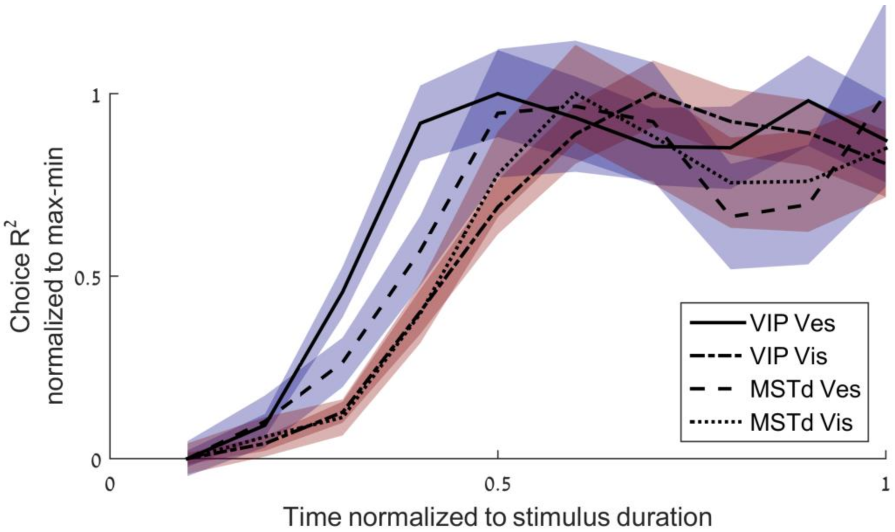

Supplementary Figure 5: Choice partial correlations (R^2) as a function of time. To compare the time course of the choice signals for visual and vestibular cues in VIP and MSTd, choice partial correlations (R^2) from Figure 4B were normalized (such that 0 and 1 represent their minimum and maximum values, respectively) and plotted on the same axis. Blue and red shaded regions reflect ± SEM of the choice partial correlation curves in the vestibular and visual conditions, respectively.

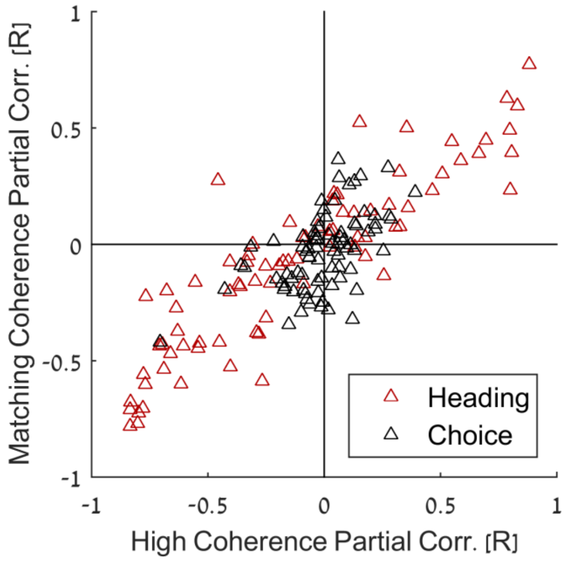

Supplementary Figure 6: Partial correlation comparison for matching vs. high coherence visual data. A comparison of the partial correlation results when using matching vs. high coherence visual stimuli, tested in the same neurons. The heading and choice partial correlations are presented in red and black, respectively.

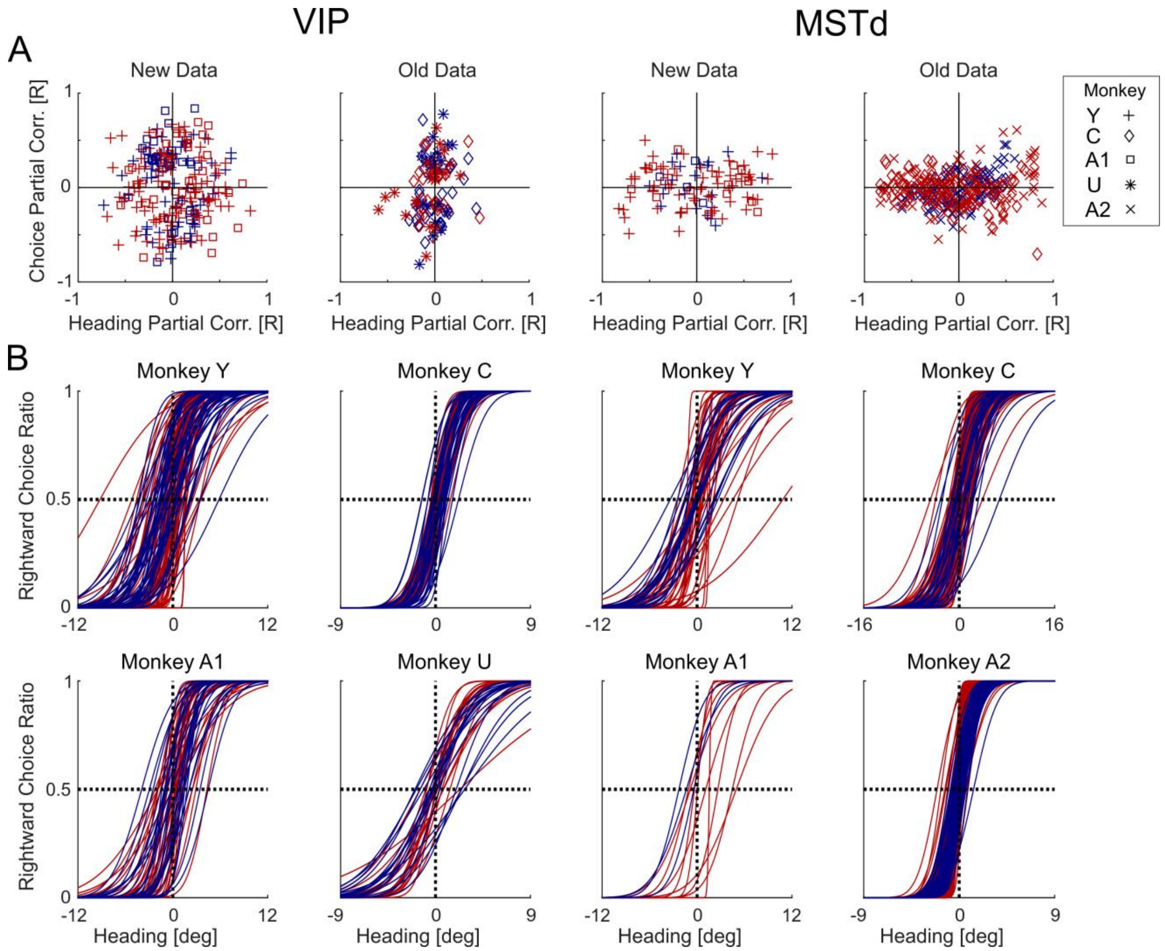

Supplementary Figure S7: Partial correlation and behavioral data for each monkey. Data were plotted separately for the 5 monkeys included in this study. (A) Heading and choice partial correlations are plotted for both the new and the old datasets from VIP and MSTd. Specific monkeys are marked by different symbols. (B) Psychometric curves from all recording sessions in A are plotted separately for the animals that were tested in each data set. The best fitting cumulative Gaussian function is shown for each psychometric function; data from the visual and vestibular conditions are shown in red and blue, respectively.
